# Development of a Dedicated X-Ray Fluoroscopic Apparatus for Therapeutic Pancreatobiliary Endoscopy: A Review

**DOI:** 10.3390/jcm14041214

**Published:** 2025-02-12

**Authors:** Taito Fukuma, Shigeto Ishii, Toshio Fujisawa, Keiko Takahashi, Tadashi Nakamura, Futoshi Shibata, Ko Tomishima, Yusuke Takasaki, Akinori Suzuki, Sho Takahashi, Koichi Ito, Mako Ushio, Muneo Ikemura, Daishi Kabemura, Hiroto Ota, Yousuke Nakai, Hirofumi Kogure, Naminatsu Takahara, Hiroyuki Isayama

**Affiliations:** 1 Department of Gastroenterology, Graduate School of Medicine, Juntendo University, Tokyo 113-8431, Japan; t.fukuma.vh@juntendo.ac.jp (T.F.); sishii@juntendo.ac.jp (S.I.); t-fujisawa@juntendo.ac.jp (T.F.); tomishim@juntendo.ac.jp (K.T.); ytakasa@juntendo.ac.jp (Y.T.); suzukia@juntendo.ac.jp (A.S.); sho-takahashi@juntendo.ac.jp (S.T.); kitoh@juntendo.ac.jp (K.I.); m-ushio@juntendo.ac.jp (M.U.); m-ikemura@juntendo.ac.jp (M.I.); d.kabemura.nc@juntendo.ac.jp (D.K.); h-ota@juntendo.ac.jp (H.O.); 2 Medical System Research & Development Center, MS R&D Department, FUJIFILM Corporation, Chiba 277-0804, Japan; keiko.takahashi.py@fujifilm.com (K.T.); tadashi.nakamura.sc@fujifilm.com (T.N.); 3 Modality Solution Division, Medical System Business Division, XR Product Marketing Group, FUJIFILM Corporation, Tokyo 107-0052, Japan; futoshi.shibata.mw@fujifilm.com; 4 Department of Gastroenterology, Graduate School of Medicine, The University of Tokyo, Tokyo 113-8655, Japan; ynakai.tky@gmail.com (Y.N.); naminatsu.takahara@gmail.com (N.T.); 5 Department of Internal Medicine, Institute of Gastroenterology, Tokyo Women’s Medical University, Tokyo 162-8666, Japan; 6 Division of Gastroenterology and Hepatology, Department of Medicine, Nihon University School of Medicine, Tokyo 101-8309, Japan; kogureh.tky@gmail.com

**Keywords:** fluoroscopy, fluoroscopic machine, pancreatobiliary endoscopy, ERCP, interventional EUS

## Abstract

In recent years, pancreaticobiliary endoscopy (PBE) has evolved to include a wide range of endoscopic procedures used to treat various diseases. Several interventional endoscopic ultrasonography procedures have been developed for conditions that cannot be treated with conventional endoscopic methods. As PBE continues to advance, it is crucial to improve fluoroscopic systems to enhance image quality, ensure patient safety, reduce radiation exposure, and ensure the operation of video-recording systems. The difficult procedures require the precise imaging of thin pancreatic/biliary branch devices, including guidewires, catheters, and stents. It is crucial to reduce noise caused by patient breathing and movement, while retaining the necessary movement in the image on the screen. A stable table is effective for ensuring the safety of patients during the procedure. A reduction in radiation exposure is important, and the flame rate conversion technique is effective. Ensuring high-quality recording is useful for the video presentation of PBE procedures. In collaboration with Fujifilm (Tokyo, Japan), we researched and developed various functions in fluoroscopic systems for PBE. In this review, we outline the requirements for fluoroscopic procedures in PBE, the evolution of technology to date, and its prospects, while also presenting the commercial equipment currently available.

## 1. Introduction

Endoscopic retrograde cholangiopancreatography (ERCP) is a diagnostic method traditionally used for biliary and pancreatic diseases. It works by cannulating the bile and pancreatic ducts through the duodenal papilla to obtain cholangiographic and pancreatographic images [1,2,3]. However, unresolved issues such as complications, including-post ERCP pancreatitis, and a relatively low success rate are significant concerns [4,5]. Recently, diagnosis for biliary and pancreatic diseases is now primarily conducted using improved imaging techniques such as multi-detector computed tomography, magnetic resonance cholangiopancreatography, and endoscopic ultrasound (EUS) [6,7,8,9,10,11,12,13,14]. The primary purpose of pancreaticobiliary endoscopy (PBE) has shifted from diagnosis to therapeutic procedures [15]. ERCP is recognized as a standard imaging technique for pancreaticobiliary procedures such as biliary and pancreatic stone removal, stenting, and more precise and invasive examinations of the bile and pancreatic ducts [16,17].

With advancements in endoscopic techniques and devices, complex procedures for conditions, previously managed surgically or percutaneously, can now be performed endoscopically [18,19,20]. These procedures include gallbladder drainage, multiple stenting for hilar bile duct cancer, and stenting for patients with surgically altered anatomies. Additionally, advancements in EUS technology led to the development of procedures that are collectively referred to as interventional EUS (I-EUS). These involve puncturing and draining the bile duct, pancreatic duct, gall bladder, and intra-abdominal abscesses via the gastrointestinal tract [21,22,23,24,25,26,27]. With the progress of I-EUS, endoscopic treatment has even become possible for cases that could not be treated with ERCP, such as those involving duodenal obstruction or surgically altered anatomies, where access to the duodenal papilla failed or proved challenging [28,29,30]. I-EUS can help to avoid pancreatitis, which is a major complication of ERCP; however, complications such as bleeding, perforation, and peritonitis remain significant concerns [31].

Precise fluoroscopic images are essential for the advancement of PBE. There are many requirements for the fluoroscopic machines used in endoscopic procedures for pancreaticobiliary diseases. In collaboration with the University of Tokyo, Juntendo University, and Fujifilm (Tokyo, Japan), we studied and developed various functions related to fluoroscopic machines for PBE. In this review article, we introduce these requirements and our solutions, showcasing currently available commercial equipment. We outline the requirements for fluoroscopic procedures in PBE, the evolution of technology to date, and the remaining challenges.

## 2. Fluoroscopy Equipment

When performing PBE, fluoroscopic equipment must have the following four functions: high fluoroscopic image quality, low radiation exposure, safe and efficient operation, and easy and efficient video recording.

Improved fluoroscopic image quality is essential for advanced PBE. Clear images of the narrow bile and pancreatic ducts, along with visible devices overlapping with intestinal gasses and vertebrae, are crucial for the success of complex procedures. It is also important to obtain clear images with reduced noise and appropriate contrasts. Because endoscopic procedures involve movement, reducing blurring is vital for safety.

There is concern that obtaining more precise fluoroscopic images may increase radiation exposure among endoscopists, medical staff, and patients. Therefore, technology is needed to address the conflicting requirements of improving image quality and reducing radiation exposure. Minimizing exposure through advanced fluoroscopic techniques and using X-ray protective equipment are potential solutions.

The safety of performing advanced endoscopic procedures on a narrow fluoroscopic table must also be considered. If the table moves during procedures, the patient may fall, or the endoscope and devices may shift. Therefore, it is preferable to adjust the fluoroscopic position by moving the tube rather than the table. Additionally, the configuration of the X-ray arm affects the ease of providing patient care for medical staff, making the layout of fluoroscopic equipment very important in endoscopic treatment.

In recent years, more research presentations on PBE have utilized videos. Videos are compelling for education, conference presentations, lectures, and papers submitted to medical journals, making video-recording functions important. Previously, video recording during procedures was performed by connecting a DVD recorder or other general-purpose recording device, which often did not capture adequately clear and detailed videos. In PBE, the simultaneous recording of fluoroscopic and endoscopic images is effective for reviews. With the rise in I-EUS procedures, it is necessary to record fluoroscopic, endoscopic, and EUS images simultaneously to accurately document the procedure. Ideally, these images should be recorded synchronously by default.

The technologies and devices developed to address the issues related to the above four fluoroscopic examination challenges are summarized in Table 1. Detailed explanations are provided in the following sections.

## 3. Efforts to Improve Image Quality of Fluoroscopy

**Figure 1:**
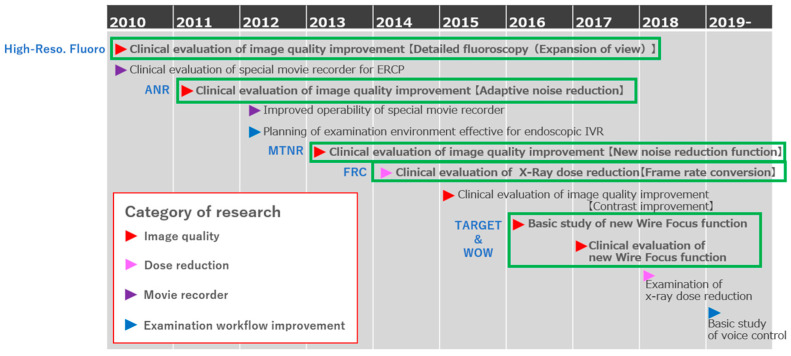
History of fluoroscopic imaging research.

Improvements were made in image quality, radiation exposure, and the operation of fluoroscopic equipment (Figure 1). In the CUREVISTA system (Fujifilm), which is commercially available worldwide, the following technologies were implemented: adaptive noise reduction (ANR), motion tracking noise reduction (MTNR), local moving device tracking noise reduction (TARGET), a high-resolution mode, and wire-optimized weighted processing (WOW) (Video S1).

### 3.1. Adaptive Noise Reduction

**Figure 2:**
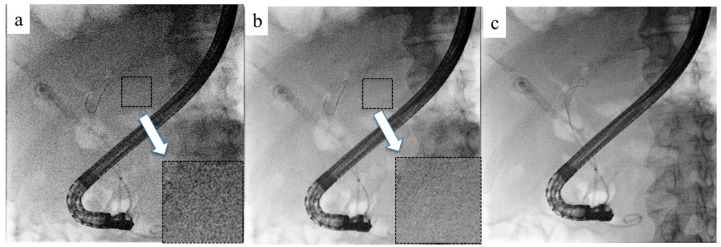
Adaptive noise reduction (ANR). (**a**) Before the introduction of ANR. Numerous noises are shown in the magnified field. (**b**) After the introduction of ANR. Noises are reduced, and the image is clearer in the magnified field. (**c**) ANR combined with a recursive filter.

Recursive filtering is a conventional technique that reduces image noise and improves observation by combining the past fluoroscopic image with the current one [32]. However, this method can cause blurring when there is motion in the subject. To address this, ANR was developed. ANR reduces image noise by appropriately switching between two noise filters using different reduction methods based on the correlations between the target pixel and its surrounding pixels (Figure 2) [33]. Combining recursive filtering with ANR effectively reduces noise. However, motion blur caused by respiratory variation is not detected, making image processing challenging in the presence of motion blur induced by respiratory variation.

### 3.2. Motion Tracking Noise Reduction

**Figure 3:**
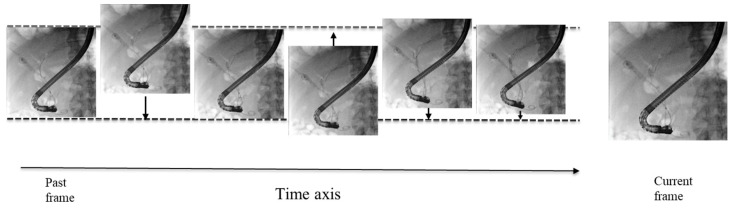
Motion tracking noise reduction (MTNR). MTNR detects global motion in consecutive fluoroscopic images with high accuracy and applies motion compensation in the direction of the arrow in the image using a recursive noise filter.

MTNR is an image processing technique that improves motion blur by applying appropriate noise reduction processing when the movement of the body and the movement of the device differ in the image (Figure 3) [34]. When motion occurs due to breathing, the image moves, causing a residual image of the devices. MTNR compensates for image movement, resulting in noise reduction.

### 3.3. Local Moving Device Tracking Noise Reduction

**Figure 4:**
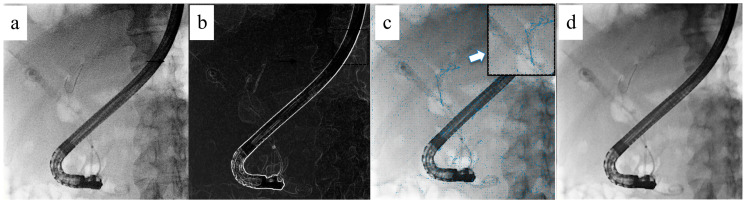
Local moving device tracking noise reduction (TARGET). (**a**) Original image. (**b**) Line detection. (**c**) Global and local motion compensation. “Local” refers to devices such as guidewires. (**d**) Final image. Noise is reduced, and the image is clearer.

When operating the guidewire, its movement may create afterimages due to the influence of breathing. To address this, the TARGET mode was developed (Figure 4). This mode captures local movements in the image, such as movements by guidewires, and provides fluoroscopic images with minimal afterimages. TARGET accurately calculates the direction and amount of movement on a pixel-by-pixel basis, tracking the object’s movement in the fluoroscopic image. This achieves effective noise reduction and the suppression of afterimages vis the recursive filter. The visibility of the device is improved, allowing the guidewire to be accurately visualized, even during manipulation or when breathing fluctuations are large.

### 3.4. Wire-Optimized Weighted Processing

**Figure 5:**
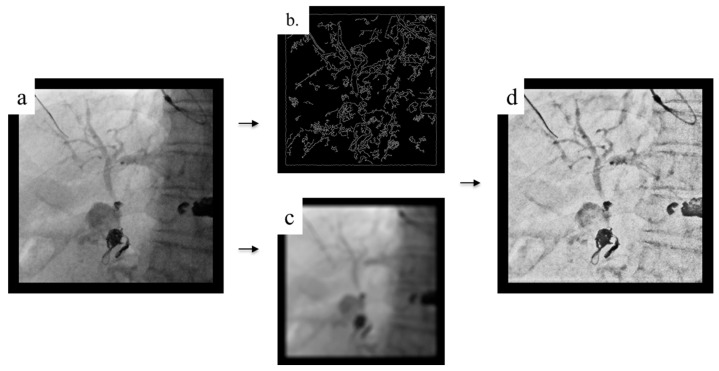
Background attenuation processing. (**a**) Original image. (**b**) Line detection. (**c**) Background image. (**d**) Final image. The background is subtracted, making devices clearly visible.

In I-EUS or pancreatic duct procedures using ERCP, the guidewire often overlaps with the vertebral body. This makes it particularly difficult to distinguish between contrast media and devices such as the guidewire, and also makes it difficult to operate the guidewire. Resolving this problem requires a fluoroscopic image that reduces the influence of vertebral bodies and improves the visibility of the guidewire. Consequently, the background attenuation processing technique was developed, which subtracts background elements such as vertebral bodies, which are similar to angiography images (Figure 5).

Background attenuation processing reduces background signals by detecting multidirectional line structures, classifying line structure signals and other background signals, and enhancing line structure signals. This enables the performance of fluoroscopy with improved visibility of the guidewire by blending the vertebrae into the background, even when it overlaps with the vertebral body or contrast medium. We named this technique wire-optimized weighted processing (WOW) because it enhances the visibility of the wire, even when overlapping with the vertebrae.

### 3.5. High-Resolution Mode

The performance of guidewires and devices used in biliary and pancreatic endoscopic treatments has dramatically improved, necessitating the production of higher-quality fluoroscopic images. Traditionally, guidewires were commonly 0.035 inches in diameter; however, thinner wires, such as 0.025 inch diameter wires, are becoming mainstream due to their ease of insertion into additional types of forceps channel. Therefore, if visibility is poor with normal fluoroscopy (3 × 3 pixels), the screen must be markedly enlarged.

In a conventional flat panel detector (FPD), the resolution is low because digital enlargement is used to expand the field of view. The high-resolution mode addresses this by enlarging the region of interest with high definition and magnification, resulting in images with higher resolutions than digitally enlarged ones. This function not only significantly magnifies the image but also allows for more precise and sharp visualization of the guidewire tip. As a result, the 0.025 inch guidewire, which was previously difficult to see, can now be seen extremely clearly in high-resolution mode.

### 3.6. Actual On-Board Function in Current Machines

Current fluoroscopic machines employ MTNR as the standard mode. High-resolution, recursive filtering with ANR, TARGET, and WOW modes is available in optional additional modes. Moreover, the frame rate conversion (FRC) is added to reduce radiation exposure. This is described in a later section.

## 4. Radiation Dose Reduction

The current radiation dose limit for the cornea is 150 mSv/year, but the recommendations of the International Commission on Radiological Protection have raised concerns among patients and medical personnel regarding medical radiation exposure [35]. In Japan, the Regulations for the Prevention of Ionizing Radiation Injury were revised in April 2021 to require an average exposure of 20 mSv/year over 5 years and no more than 50 mSv/year in any single year. Therefore, measures to reduce radiation exposure are urgently needed. The three principles of radiation protection are time, distance, and shielding.

Time: It is important to minimize the fluoroscopy time [36]. The endoscopist and the person controlling the fluoroscopic machine should communicate effectively to avoid unnecessary fluoroscopy, thereby reducing radiation exposure.

Distance: Scattered radiation is attenuated inversely with the square of the distance [37]. Although it is difficult for the endoscopist to maintain distance during procedures, non-endoscopists should stay as far from the fluoroscopy equipment as possible when not needed.

**Figure 6:**
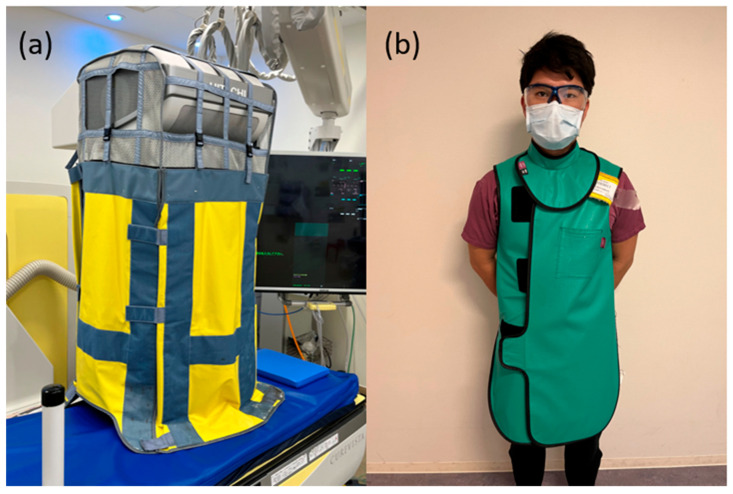
Shielding. (**a**) Curtain-type protective covers provide radiation shielding in all directions. (**b**) Protective clothing and cover collars with a lead equivalent of 0.25 mm, providing approximately 92–93% shielding effectiveness.

Shielding: Shields, protective clothing, collars, and eyewear are available as protective measures. The challenge with shielding is that it only protects in one direction, necessitating additional protection outside the shielded field. Curtain-type protective covers have recently been developed that attach to transparent tubes, providing all-direction shielding (Figure 6) [37,38]. Protective clothing and collars are available in three lead-equivalent weights (0.25, 0.35, and 0.50 mm), with 0.25 mm providing a shielding effect of 92–93% for the X-rays commonly used in ERCP (Figure 6) [39,40,41]. Higher lead-equivalent values offer greater protection but are heavier and increase the burden on the endoscopist. Radiation protection glasses reduce the dose by <50% [42]. Facilities using over-tubes should consider protective eyewear. In addition to these measures, fluoroscopic equipment was developed to reduce radiation exposure.

By integrating these principles and advancements, radiation exposure can be effectively managed, ensuring the safety of both patients and medical personnel.

### 4.1. Diagnostic Reference Level (DRL)

The DRL is a standard for the use of radiation introduced by the International Commission on Radiological Protection in 1996 [43]. The DRL concept aims to keep radiation use as low as achievable by setting standard doses for specific examinations. Because DRLs differ by country for the same examination, they are periodically updated by each country’s radiological society.

In the REX-GI study conducted in Japan in 2019, data on radiation doses for various procedures, such as ERCP, I-EUS, balloon-assisted enteroscopy, gastrointestinal stent placement, and ileus tube placement, were collected from multiple facilities across Japan [44,45]. Analysis using ERCP was further subdivided into common bile duct stones, proximal malignant biliary obstruction, distal malignant biliary obstruction, and pancreatic diseases. In particular, it was shown that radiation exposure was higher in cases of proximal malignant biliary obstruction.

It is anticipated that in the 2025 Japanese DRL revision, target values will be set for each subgroup, and each facility will need to strive to stay below these target values.

### 4.2. Frame Rate Conversion

**Figure 7:**
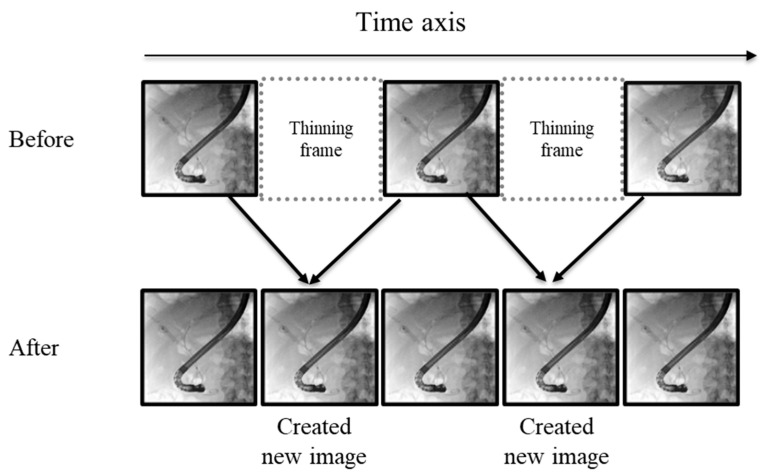
Frame rate conversion (FRC). FRC generates interpolated frames from two consecutive fluoroscopic images, improving the quality of the fluoroscopic image by increasing the frame number without increasing the radiation amount.

High-resolution and TARGET modes have significantly improved image quality, but there have been concerns about increased radiation exposure due to the increased number of image frames [46]. The frame rate conversion (FRC) technique addresses this by creating interpolated images from previous and subsequent frames (Figure 7). This results in smoother fluoroscopic images due to the increased frame number while keeping the radiation dose at half.

## 5. Operation of Machines for Patient Safety and Procedure Efficiency

With the introduction of the FPD for X-ray fluoroscopy in 2001, both image quality and radiation exposure improved [47,48]. Additionally, the movable range of the X-ray tube arm became wider, the blind area became smaller, and the lowering of the examination was enabled. However, previous X-ray fluoroscopic systems required us to move the fluoroscopy table to the left or right, or to lift and move the patient to align the fluoroscopy position with the target area, necessitating further improvements.

**Figure 8:**
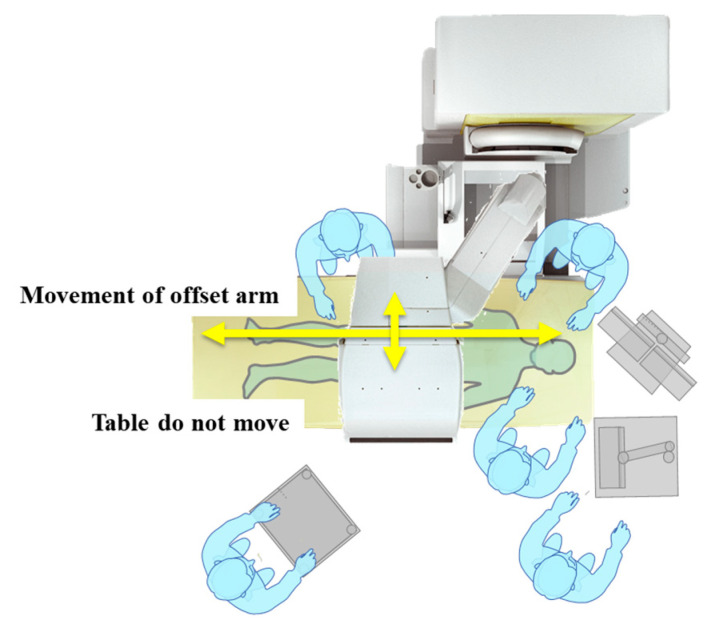
Current operation of fluoroscopic machine: CUREVISTA (Fujifilm). The offset open design allows for a large workspace on the far side of the table, making it easy to provide support to the patient. The fluoroscopy system can also be moved horizontally and vertically (in the direction of the arrows in the image), allowing procedures to be performed without moving the patient.

The CUREVISTA system (Fujifilm) addresses these challenges by allowing the field of view to move in both vertical and horizontal directions using a two-way arm that moves the imaging system (both the tube and FPD) (Figure 8). This enables safe procedures without moving the patient, even when an endoscope is inserted during ERCP or a needle is inserted during I-EUS or percutaneous procedures. The CUREVISTA system is also a safe and reliable tool.

The offset arm and table and the open design of the CUREVISTA provide sufficient space on both sides of the table and around the patient’s head (Figure 8). This allows the endoscopist and caregiver easy access from both sides of the patient and enables the placement of multiple endoscopic systems at the side of the patient’s head for procedures such as peroral cholangioscopy.

## 6. Recording System

Previously, fluoroscopic videos were recorded with a reduction in the number of scanning lines from 1000 to 500, resulting in lower definition. To address this, we developed the VC-1000 recording device, manufactured by Fujifilm, which maintained 1000 scanning lines for high-definition recording. The VC-1000 can record endoscopy and fluoroscopy images simultaneously.

**Figure 9:**
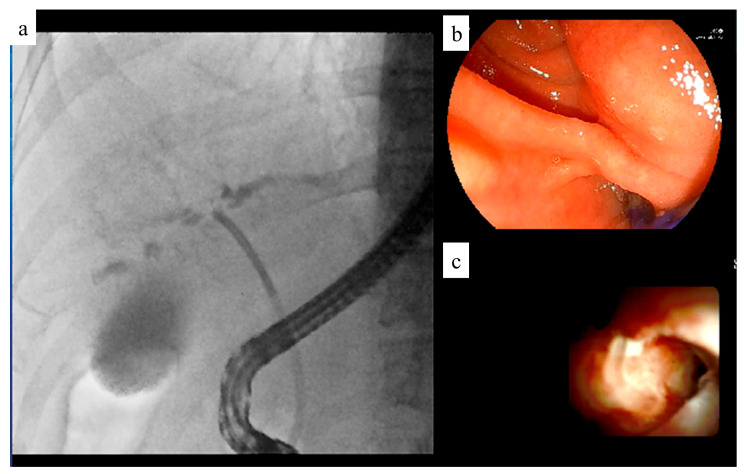
Recording system. This system can record multiple images simultaneously. (**a**) Fluoroscopic image. (**b**) Endoscopic image. (**c**) Peroral cholangioscopic image.

The upgraded VC-2000, which can record up to four systems of video information simultaneously, has since been introduced (Figure 9). The VC-2000 can simultaneously record endoscopic, fluoroscopic, and EUS images as well as images from video cameras installed in the examination room. This allows the endoscopist to review the operation images after the examination. The ability to review video images recorded simultaneously facilitates communication between instructors and junior endoscopists.

## 7. Future Perspective

As PBE becomes more advanced, further improvement in fluoroscopic equipment is needed. In addition to improving image quality, by creating a fusion image that combines 3D virtual fluoroscopic images generated from pre-endoscopic CT or MRI with fluoroscopic images, it becomes easier to understand the positional relationships between the target area, the approach route, and structures such as the vertebral body. This allows for more precise endoscopic procedures. Moreover, efficiency may be enhanced by technology that allows the patient to change the fluoroscopy mode, frame rate, and fluoroscopy position via vocal commands. If technology were available that allowed changes to fluoroscopy mode, frame rate, and fluoroscopy position through voice commands from the patient, this would enhance efficiency in scenarios where both hands and feet are occupied during scope operation or when performing procedures with a small team. Enabling diagonal movements similar to those of under-table fluoroscopy devices would allow angle adjustments without moving the patient, facilitating better observation of overlapping intrahepatic bile ducts and improving clarity in distinguishing anterior and posterior structures.

Notably, however, occupational exposure to scattered X-rays among medical personnel is increasing. Therefore, visualizing scattered X-rays with a dose map, which makes these invisible rays understandable, will help to raise awareness of occupational exposure among medical personnel. Additionally, enabling the development of technology that allows for easy restriction of the irradiation field in vertical and horizontal directions would make it possible to only observe the necessary examination area, further reducing radiation exposure.

## 8. Limitation

The limitations of this review include the scarcity of reports discussing the evolution of fluoroscopic technology, making comparisons with existing studies challenging. Additionally, since this review focuses on the evolution of fluoroscopic technology in Japan, it does not encompass the global development of fluoroscopic techniques, which is also a noted limitation.

## 9. Conclusions

With the advancement of endoscopic pancreatobiliary procedures, obtaining more precise images without increasing radiation exposure is essential. Ensuring the safety of both patients and medical staff during endoscopic procedures is also crucial. The current fluoroscopic technology was developed based on the needs of endoscopists and via technology provided by companies. On the other hand, expectations for minimally invasive treatments remain high, and with the anticipated increasing complexity of therapeutic procedures, the need for prolonged or multiple sessions is expected to grow. As a result, further improvements in image quality and radiation exposure reduction are more crucial than ever. It is important for endoscopists and companies to continue working together to improve fluoroscopic devices in the future.

## Figures and Tables

**Table 1:** Requirements for and solutions of fluoroscopic machines.

Improvement in image quality
Precise and magnifying image
High-resolution mode
Noise reduction
Adaptive noise reduction (ANR)
Clear movement image and deletion of remaining image
Motion tracking noise reduction (MTNR)
Local moving device tracking noise reduction (TARGET)
Subtraction of the spine, injected contrast medium, and GI tract gas
Wire-optimized weighted processing (WOW)
Reduction in radiation exposure
Shield
Reduction in the flames
Patients’ safety during the procedure
Large table
Stable table (movement of the radiation system)
VTR recording systems
High-resolution image
Picture in picture
Convenient recording system

## Data Availability

No new data were created or are available for this study. The analysis and conclusions presented in this paper are based on previously published data and existing information.

## Supplementary Materials

The following supporting information can be downloaded at https://www.mdpi.com/article/10.3390/jcm14041214/s1, Video S1: Comparison of fluoroscopic technology before and after implementation.
